# Rapamycin Alleviates the Symptoms of Multiple Sclerosis in Experimental Autoimmune Encephalomyelitis (EAE) Through Mediating the TAM-TLRs-SOCS Pathway

**DOI:** 10.3389/fneur.2020.590884

**Published:** 2020-11-27

**Authors:** Xiao-ling Li, Bo Zhang, Wei Liu, Meng-jiao Sun, Ya-lan Zhang, Hui Liu, Man-xia Wang

**Affiliations:** ^1^Department of Neurology, Lanzhou University Second Hospital, Lanzhou, China; ^2^Department of Cardiology, The First Hospital of Lanzhou University, Lanzhou, China

**Keywords:** multiple sclerosis, rapamycin, GAS6, TAM, TAM-TLRs-SOCS pathway

## Abstract

Multiple sclerosis (MS) is an inflammatory demyelinating disease of the central nervous system (CNS). Our research aimed to find an immunomodulatory therapy for MS. An experimental autoimmune encephalomyelitis (EAE) mouse model of MS was established induced with the syntheticmyelin oligodendrocyte glycoprotein peptide 35-55 (MOG35-55). Fifty C57BL/6 mice were randomly divided into the Normal group, EAE group, and Rapamycin group (EAE mice treated with three different doses of rapamycin). Hematoxylin and eosin staining and Weil myelin staining were performed on the brain tissues of mice after 21 days post-immunization. The protein expression of Gas6, Tyro3, Axl, Mer in paraventricular tissues were analyzed by immunohistochemistry. The mRNA and protein expression of Gas6, Tyro3, Axl, Mer, SOCS1, SOCS3, Toll-like receptor (TLR) 3, and TLR4 were detected by quantitative real-time PCR (qRT-PCR) and Western blot, respectively. An enzyme-linked immunosorbent assay (ELISA) was used to detect the secretion of the inflammatory factors IFN-γ and IL-17. Rapamycin treatment could ameliorate the behavior impairment in EAE mice induced by MOG35-55. The expression of Gas6, Tyro3, Axl, Mer, SOCS1, and SOCS3 were decreased in EAE mice at 21 days post-immunization, while the expression of Gas6, Tyro3, Axl, and Mer in rapamycin group was higher than that in EAE group. It was accompanied by an increase in anti-inflammatory proteins SOCS1 and SOCS3, a decrease in the inflammatory proteins TLR-3, TLR-4 and in the amount of IFN-γ, and IL-17. Rapamycin injection relieved the nerve function of and the loss of myelin sheath in the EAE mice, mainly through mediating the TAM-TLRs-SOCS signaling pathway to regulate natural immunity.

## Introduction

Multiple sclerosis (MS) is a demyelinating autoimmune disease of the central nervous system (CNS). Remission-relapse is a key clinical characteristic of the disease (1). Currently, the treatment of MS includes acute episode therapy and immunomodulatory therapy. Nevertheless, reducing the recurrence rate remains a significant challenge to the treatment of MS. Immunomodulatory therapies of the first generation, including interferon-β (IFN-β) and glatiramer acetate, have become the standard of care in relapsing-remitting MS (2). However, there are not enough immunomodulatory drugs such as trifluramine and IFN-β to control the recurrence of MS, and they are considerably high cost (3). Thus, new drugs for MS therapy still need to be developed.

Rapamycin is a macrolide immunosuppressant isolated from *Streptomyces hygroscopicus* that has been found to produce certain neuroprotective, neurotrophic, and anti-aging effects (4, 5). It has been shown that rapamycin could improve the treatment of MS as the symptoms of patients with relapse-remission MS improved significantly after treatment with rapamycin for 6 months (6). Rapamycin is considered a classical autophagy inducer and a specific inhibitor of mammalian rapamycin target protein (mTOR) (7–9), and its effects have been studied in the experimental autoimmune encephalomyelitis (EAE) animal model of autoimmunity that shares several clinical and pathologic features with MS (10, 11). However, the mechanisms which rapamycin inhibits the innate immunity and inflammatory demyelination have not been established.

The TAM family of receptor tyrosine kinases (RTKs), including Tyro3, Axl, and Mer, are widely expressed in mammalian immune, blood, reproductive, and nervous systems (12). Furthermore, the TAM family has been implicated in the regulation of MS. Single knockout mice that lacked *Axl* (Axl^−/−^) were induced with syntheticmyelin oligodendrocyte glycoprotein peptide 35-55 (MOG35-55), which increased inflammation and reduced the clearance of debris following demyelination (13–15). In addition, during MOG 35-55-induced EAE it has been reported that the TAM receptor ligand, Gas6, dampened the inflammatory response and preserved CNS function (16).

In this study, we aimed to establish a mechanism for the involvement of rapamycin in innate immune regulation by investigating its effect on Gas6/TAM signaling through dynamic changes in the members of the Gas6/TAM-TLRs-SOCS pathway. It will provide a basis understanding for rapamycin in the immunomodulatory therapy of MS.

## Materials and Methods

### Animal Preparation

Fifty female C57BL/6 mice (aged 6–8 weeks, 16–18 g) were purchased from the Animal Research Center of Gansu University of Traditional Chinese Medicine and reared in the Animal Laboratory of Gansu University of Traditional Chinese Medicine (Lanzhou, Gansu Province). Mice were maintained in a controlled 12:12 h light/dark environment and provided food and water. For the study, mice were randomized into a normal control group, EAE model group, and EAE groups that were treated with a low, medium, or high dose of rapamycin with 10 mice per group. The study was approved by the Ethics Committee of the Second Hospital of Lanzhou University.

### EAE Model Preparation and Treatment Evaluation

EAE was actively induced in mice using MOG35-55(Sigma-Aldrich) as previously described (5). In brief, MOG35-55 was diluted to 10 mg/mL in 0.9% physiological saline and combined with an equivalent volume of complete Freund's adjuvant (CFA, Chondrex) and tuberculin H37Ra (DIFCO) at a concentration of 4 mg/mL. The solution was pumped into an oil-in-water form by syringe to prepare an induction antigen emulsion. Mice were subcutaneously injected with 0.1 mL of the emulsifier at 4 points on both sides of the spine and then intraperitoneally injected with 0.5 mL of pertussis toxin (PT, American LBL Company) twice at 0 and 48 h post-immunization. One week later, the mice received a booster injection of the MOG35-55 solution.

### Rapamycin Treatment

EAE mice were intraperitoneally injected once a day with distilled water (negative control) or rapamycin (Sigma-Aldrich) dissolved in distilled water after the 10 days immunization with MOG35-55. The dose was selected based on previous reported studies. The optimal dose from different studies was found to be 1 mg/ kg (17) 2 mg/ kg (18) and 2.5 mg/ kg (19). The treatment mice were administered a low dose (1 mg/kg, body weight), medium dose (2 mg/kg), or high (2.5 mg/kg) dose of rapamycin. Mice in the Normal group were injected with distilled water as a vehicle control.

### Clinical Assessment

Mice were examined daily after injection, and the clinical indications were scored using the following five-point scale: 0, no clinical signs; 1, limp tail; 2, impaired righting; 3, paresis of one hind limb; 4, paresis of two hind limbs; or 5, moribund. Accumulative clinical score was calculated for each mouse by adding the daily scores from the day of onset until the end of the treatment. Mice were weighed daily from the first day after immunization by two observers using a double-blind method.

### Histological Evaluation

The normal control group, EAE group, and the middle-dose rapamycin group at 21 days post immunization were selected for histological evaluation. Five mice were chosen at random from each group and anesthetized with 10% chloral hydrate (10 mg/kg) and decapitated after blood collection. The brains were dissected, numbered, and fixed in 10% formalin in phosphate-buffered saline. The tissue samples were routinely treated and embedded in paraffin. Coronal sections (5-μm) were made for brain specimens, haematoxylin and eosin (HE) stained sections were used to assess inflammatory cell infiltration, and Weil myelin stained paraffin sections were used to evaluate remyelination. NISE lements BR30 image processing software was used for image analysis. Inflammatory cell infiltration and demyelination in the brain were observed.

### Immunohistochemistry

Gas6, Mer, Axl, and Tyro-3 proteins in brain tissues were measured by immunohistochemistry of 5-μm tissue sections. Anti-Myelin Basic Protein antibody (MBP antibody sc-271524, 1:500, Santa Cruz Biotechnology, Dallas, TX, United States) labels myelin which is the most abundant protein in the myelin membrane. For this, sections were deparaffinized, rehydrated, and treated with 3% (v/v) H_2_O_2_ in methanol for 30 min, followed by blocking with 5% (w/v) fat-free milk for 1 h. Subsequently, they were incubated with a primary antibody (anti-Mer, ab79223; anti-Axl, ab219651; or anti-Tyro 3, ab109231; anti-FOXP3 antibody, ab20034, 1:100, Abcam, UK) at 4°C overnight. After washing, bound antibodies were detected with biotin-labeled secondary antibodies using the ABC kit, visualized with diaminobenzidine, and examined by light microscopy (Thermo Fisher Scientific, USA). Immunostained slides were independently scored by at least three investigators and the mean of the results was calculated.

### qRT-PCR

The genes *Gas6, Tyro3, Axl, Mer, SOCS1, SOCS3, TLR-3*, and *TLR-4* of mice in the Normal group, EAE group, and Rapamycin group were collected. Total RNA was extracted by combining 70-mg of brain tissue with 1 mL Trizol reagent. Then total RNA was reverse-transcribed into cDNA by PrimeScript RT reagent Kit (TaKaRa, Dalian, China). The All-in-One qPCR mix and validated primers (Fulengen) kit was used for the qRT-PCR assay performed with a LightCycler (Roche). The reaction mixture contained the reaction system (20 μL), 2 x All-in-One qPCR Mix (10 μL), cDNA (2 μL), All-in-One qPCR Primer 2 μL, and sterilized distilled water supplemented to 20 μL. The cycling conditions were as follows: 1 cycle at 95°C for 10 min and 40 cycles of 95°C for 10 s, 60°C for 20 s, and 72°C for 15 s. Dissolution curves were produced at 72–95°C for 6 s and 25°C for 30 s. GAPDH was used as internal controls of mRNA. All data were analyzed using the 2^−ΔΔ^CT method. Triplicates of the assays were repeated for ≥3 times. Primers were listed in Table 1.

**Table 1:** Primers sequences.

**Name of primer**	**Sequences (5^**′**^-3^**′**^)**
*Gas6*-F	CTTGCATCAGACGGCCAGAC
*Gas6*-R	CCTCATCGCAGAGGCAAGAGTA
*Tyro3*-F	CAGGGCTAAAGGTCGTCTCC
*Tyro3*-R	ATGTCCACCATGAACCGGAC
*Axl*-F	GGGGATTACTACCGCCAAGG
*Axl*-R	TCTCCCACATTGTCACACCG
*Mer*-F	AAACTGCATGTTGCGGGATG
*Mer*-R	ATGGCGATCCACTTCACAGG
*SOCS1*-F	CACTCACTTCCGCACCTTCC
*SOCS1*-R	GTCCCCAATAGAAGCCGCAG
*SOCS3*-F	CCTTTTCTTTGCCACCCACG
*SOCS3*-R	AGAGAGGTCGGCTCAGTACC
*TLR-3-*F	TTGTCTTCTGCACGAACCTG
*TLR-3-*R	GGCAACGCAAGGATTTTATT
*TLR-4-*F	AGACCTGTCCCTGAACCCTAT
*TLR-4-*R	CGATGGACTTCTAAACCAGCCA
GAPDH-F	CTTGGGCTACACTGAGGACC
GAPDH-R	CATACCAGGAAATGAGCTTGAC

### Western Blot

Brain tissues were dissected from mice (*n* = 5) in the Normal group, EAE group, and Rapamycin group on day 21 post-immunization and frozen immediately in liquid nitrogen and homogenized with 500 μL of lysate. Total protein concentration was determined using the Coomassie brilliant blue. Total protein was separated by 10% SDS-PAGE gel electrophoresis and transferred onto a PVDF membrane. Membranes were blocked in 5% skimmed milk in PBS at room temperature for 2 h before incubation with a primary antibody at 4°C overnight. The PVDF membrane was incubated in peroxidase-labeled secondary antibody (anti-rabbit IgG, 1:2,000, A6154MSDS, Sigma-Aldrich, USA) for 2 h at room temperature and ECL luminescent solution was added. The protein bands were visualized with ECL system (Thermo, USA), and images were analyzed using Image J software.

### Enzyme-Linked Immunosorbent Assay (ELISA)

EAE mice were immunized on 21 days, and five mice per group were anesthetized with chloral hydrate. Blood samples were randomly collected from mice (*n* = 5) and serum was separated. The levels of IFN-γ and IL-17 were determined using an ELISA according to the manufacturer's instructions (BD Biosciences, USA).

### Statistical Analysis

All data were analyzed using SPSS version 22.0 and GraphPadPrism7 and represented as the mean ± SD. A repeated-measures ANOVA was used to identify significant differences at different time points within a group. The Student *t*-test was used to compare the data between two groups at the same time point if the data conformed to a normal distribution. If data did not conform to normal distribution the rank-sum test was used. Differences were considered statistically significant at *P* < 0.05.

## Results

### Effect of Rapamycin on EAE Mice Clinical Scores

**Figure 1:**
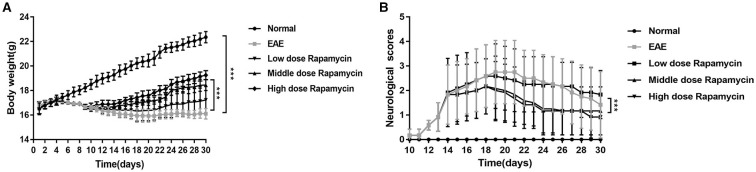
Effect of Rapamycin on EAE mice clinical scores. **(A)** Body weight of the Normal group, the EAE group and rapamycin (a low, medium, or high dose of rapamycin) group during 30 day post immunization clinical follow-up. **(B)** The neurological scores of the Normal group, the EAE group and rapamycin group during 30 day post immunization clinical follow-up. ***P < 0.001 vs. EAE group.

There was a significant difference in body weight between the EAE group and Normal group (*P* < 0.001). However, the body weights of low-dose, medium-dose, and high-dose rapamycin groups were increased when compared with EAE group during study period (30 days) (*P* < 0.001, Figure 1A). Clinical scores are one of the validated behavioral methods used to evaluate the symptoms of EAE in mice. Following induction of EAE with MOG35-55, the symptoms of EAE mice with and without rapamycin treatment were compared by using clinical score testing. The EAE and rapamycin groups achieved a higher clinical score than the Normal group stared at day 14 (*P* < 0.05). The neurological scores of low-dose, medium-dose, and high-dose rapamycin groups were compared and a significant lower was identified between the low-dose group and the medium as well as high-dose groups (*P* < 0.01). No significant difference was found between the medium-dose group and the high-dose group (Figure 1B). These results demonstrated that rapamycin treatment can ameliorate the behavior impairment in EAE mice induced by MOG35-55. In addition, a medium dose of rapamycin was selected to inject intraperitoneally into EAE mice for further study.

### Effect of Rapamycin on Pathological Changes of Brain Tissue in EAE Mice

**Figure 2:**
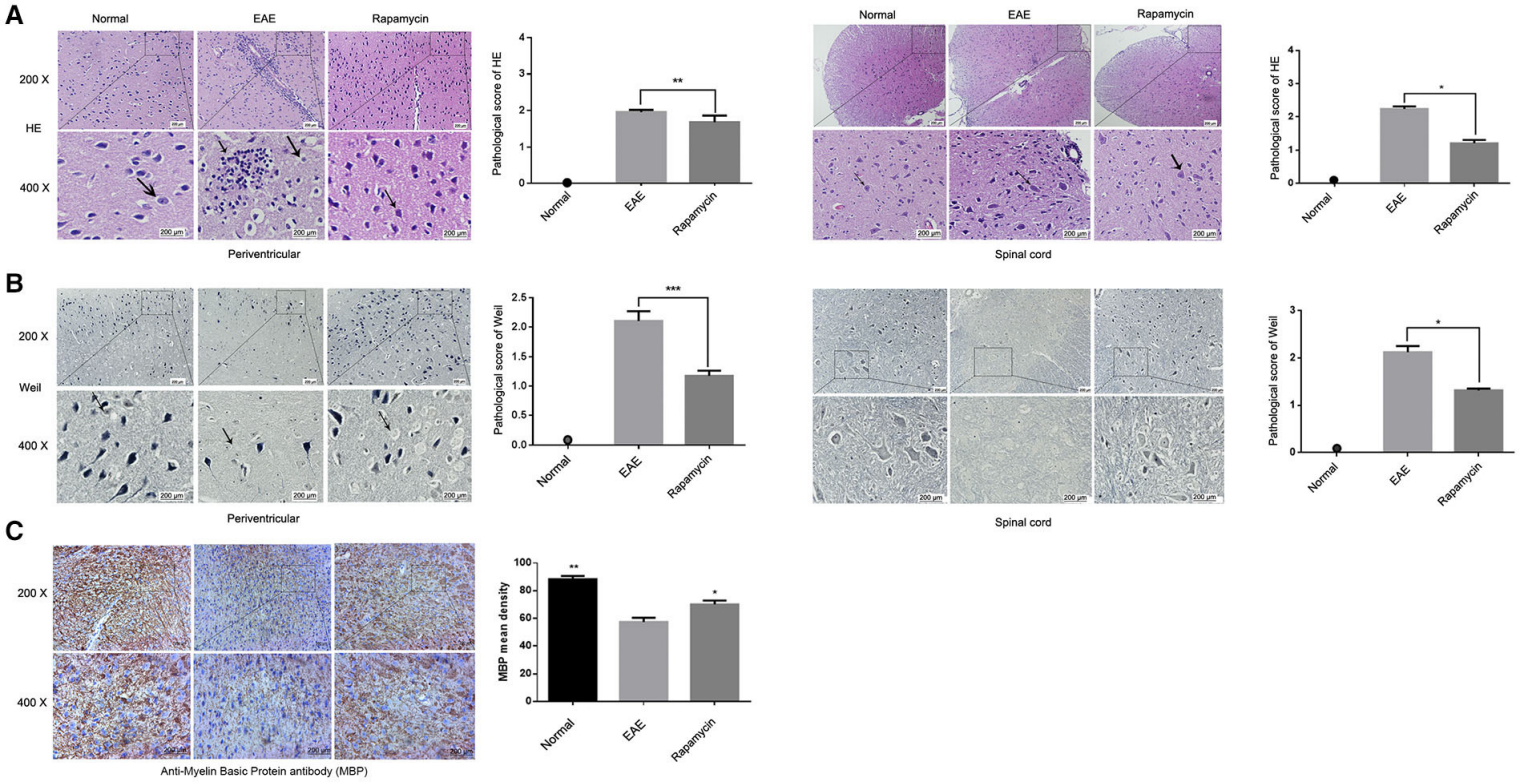
Effect of Rapamycin on pathological changes of tissue in EAE mice. **(A)** HE staining of the tissue in the periventricular and spinal cord at 21 day post immunization (left: Normal group, mid: EAE group, right: Rapamycin group, 200x,400x magnification). **(B)** Weil staining of tissue in the periventricular and spinal cord at 21 day post immunization (left: Normal group, mid: EAE group, right: Rapamycin group, 200x, 400x magnification). **(C)** Anti-Myelin Basic Protein antibody (MBP antibody) has been detected in spinal cord to demonstrate the retention of more myelin (left: Normal group, mid: EAE group, right: Rapamycin group, 200x,400x magnification). *P < 0.05, **P < 0.01, ***P < 0.001.

Pathological changes in the brain were evaluated in the Normal group, EAE group, and Rapamycin group after 21 days post immunization. The main pathological changes were increased inflammatory cell infiltration and demyelination (Figure 2). HE staining showed that the tissues of mice in the EAE group have a large number of inflammatory cells, inflammatory cells mainly gathered around the vascular, forming the structure like “cuff.” Inflammatory cells were significantly reduced in Rapamycin group when compared with the EAE group (*P* < 0.05, *P* < 0.01, Figure 2A). Weil staining of the tissues dyed the myelin sheath a blue-black color. The myelin sheath lesions were characterized by dilatation of the myelin sheath, focal cavities, rupture of many nerve fibers and membranes, and the disappearance of axons. A large number of inflammatory and demyelination were obvious in the EAE group, rapamycin treatment could reduce the number of inflammatory (P < 0.05, P < 0.001, Figure 2B). Furthermore, for the spinal cord, areas (pixels) of positive MBP immunostaining were measured and showed that the retention of more myelin in the rapamycin group of mice (*P* < 0.05, Figure 2C).

### The Expression of GAS6/TAM Receptor Was Analyzed by Immunohistochemistry

**Figure 3:**
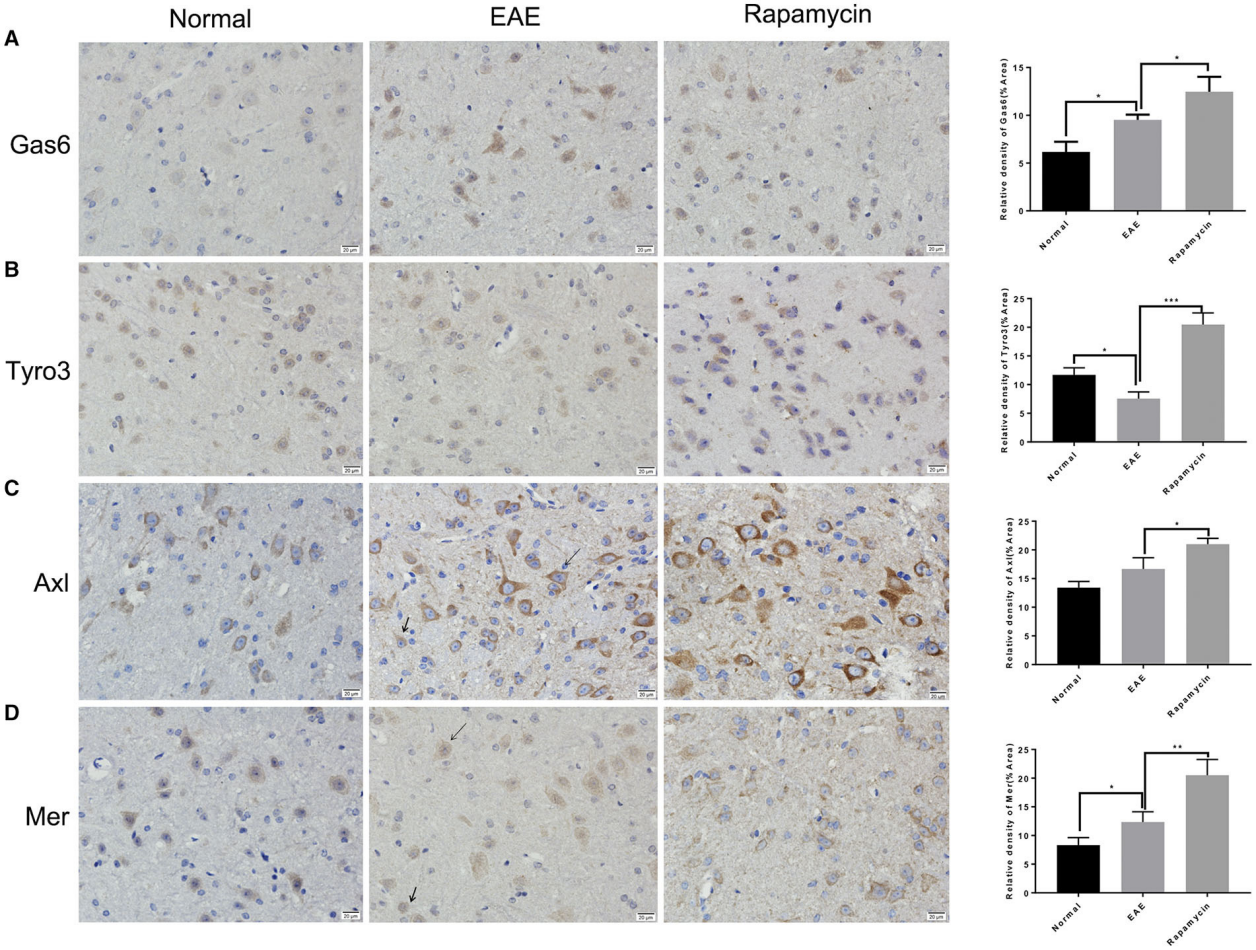
The expression of GAS6/TAM receptor (Tyro3, Axl, and Mer) was analyzed by immunohistochemical analysis. Immunohistochemical analysis of the protein level of GAS6 **(A)**, Tyro3 **(B)**, Axl **(C)**, and Mer **(D)** in the periventricular at 21 day post immunization (left: Normal group, mid: EAE group, right: Rapamycin group, 400x magnification). Arrow heads indicated the neurons and microglia. *P < 0.05, **P < 0.01, ***P < 0.001.

Paraffin sections of brain tissues in the periventricular were prepared from the Normal group, EAE model group, and the Rapamycin group at 21 days post immunization. Immunohistochemical staining showed that the protein levels of Gas6, Tyro3, Axl, and Mer in the Rapamycin group were higher than the EAE group (*P* < 0.05, *P* < 0.01, *P* < 0.001, Figures 3A–D). Meanwhile, we found that Gas6 and Tyro3 were mainly expressed in the neurons of the paraventricular tissues. Axl and Mer were expressed in neurons and microglia in paraventricular tissues.

### Rapamycin Up-Regulated the TAM Family but Down-Regulated TLR

**Figure 4:**
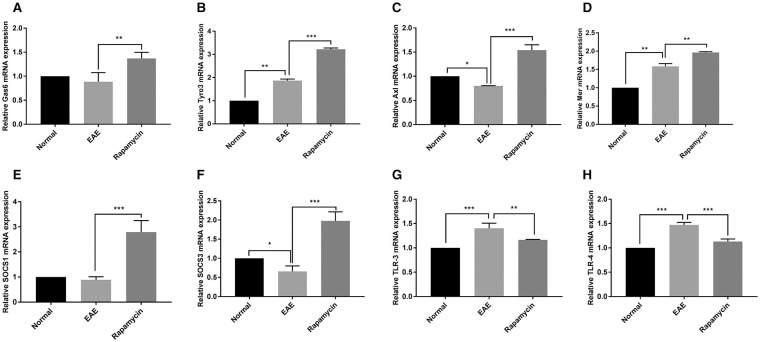
The mRNA expression of Gas6 **(A)**, Tyro3 **(B)**, Axl **(C)**, Mer **(D)**, SOCS1 **(E)**, SOCS3 **(F)**, TLR-3 **(G)**, and TLR-4 **(H)** in c at 21 days post immunization. *P < 0.05, **P < 0.01, ***P < 0.001.

The demyelination of paraventricular tissue is atypical pathological feature of MS; therefore, we selected paraventricular tissues for further analysis the effect of rapamycin using qRT-PCR. In the EAE group, the expression of Tyro3, and Mer at 21 days post immunization was significantly increased, the expression of Axl at 21 days post immunization was significantly decreased when compared with the Normal group (*P* < 0.05). In contrast, when compared to the EAE group, the expression of Gas6, Tyro3, Axl, and Mer was increased in Rapamycin group (*P* < 0.01, *P* < 0.001, Figures 4A–D). We also measured the expression of SOCS1 and SOCS3, which act downstream of the TAM receptor to inhibit the innate immune response. Although when compared with the Normal group, SOCS3 expression was decreased in EAE mice at 21 days, the expression of SOCS1 and SOCS3 in Rapamycin group was higher than the EAE group (*P* < 0.001, Figures 4E,F). In addition, in EAE group, TLR-3 and TLR-4 expression were more abundant than in the Normal group (*P* < 0.001), which is consistent with the role of TLRs as key receptors in the innate immune response. However, the expression of TLR-3 and TLR-4 was significantly lower in the Rapamycin group compared with the EAE group at 21 days (*P* < 0.01, *P* < 0.001, Figures 4G,H).

**Figure 5:**
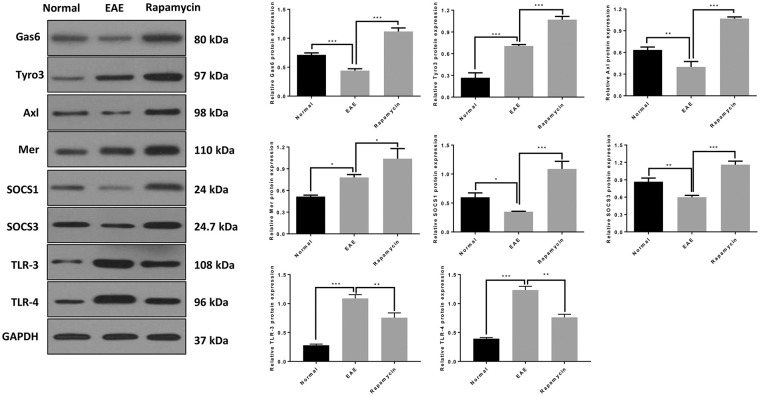
The protein expression of Gas6/TAM, SOCS1, SOCS3, TLR-3, and TLR-4 in the Normal group, EAE group and Rapamycin group at 21 days post immunization. *P < 0.05, **P < 0.01, ***P < 0.001.

The Western blot results showed that Gas6, Tyro3, Axl, Mer, SOCS1, and SOCS3 protein levels in the Rapamycin group were significantly higher at 21 days post immunization, compared with the EAE group (*P* < 0.01, *P* < 0.001). Those findings were consistent with the qRT-PCR results. Furthermore, the protein levels of TLR-3 and TLR-4 were also lower in the rapamycin group than in the EAE group at 21 days (*P* < 0.01, Figure 5).

### IFN-γ and IL-17 Were Increased in EAE Mice but Inhibited by Rapamycin Treatment

**Figure 6:**
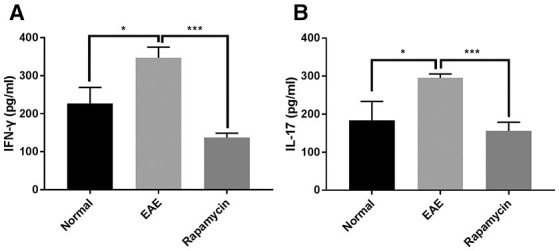
Levels of IFN-γ and IL-17 were increased in EAE mice but inhibited by rapamycin treatment. Determination the levels of IFN-γ **(A)** and IL-17 **(B)** by ELISA analysis in the Normal group, EAE group and Rapamycin group at 21 days post immunization. *P < 0.05, ***P < 0.001.

Cytokines including IFN-γ and IL-17 are associated with the regulation of MS regulation via mechanisms such as destruction of the myelin sheath or promotion of the inflammatory response. The results of the ELISA showed that the levels of IFN-γ and IL-17 were significantly higher than in the Normal group at 21 days post immunization (*P* < 0.05). However, rapamycin treatment could decrease the levels of IFN-γ and IL-17 in EAE mice (*P* < 0.001, Figures 6A,B).

## Discussion

In this study, we found that rapamycin could regulate the TAM-TLRs-SOCS pathway by up-regulating the TAM receptors (Gas6, Tyro3, Axl, and Mer) to play an immunosuppressive effect in the inflammatory demyelination of EAE mouse brain tissue.

To investigate the role of rapamycin for MS, an EAE animal model of autoimmunity was established using MOG35-55, which induced inflammation in the mice. EAE was induced with MOG35-55 in C57B6/L mice, which is the commonest animal model used to study MS (20). Then, clinical score assessment, a widely accepted behavioral method for evaluating EAE symptoms, was used to observe the variation among the mice (21). In our study, EAE mice presented weakness in the tail and hind limbs with a loss of appetite, weight loss, non-smooth fur, and neurological deficits. The results of subsequent clinical score testing demonstrated that the mice treated with rapamycin achieved markedly better scores relating to body weights and neurological scores compared with EAE mice. Subsequently, the variation of pathology, including HE staining and Weil myelin staining, was assessed in the brain. HE staining and Weil staining showed that a large number of inflammatory cells and demyelination were obvious in the EAE group, while rapamycin treatment could reduce the number of inflammatory cells and myelin damage.

Previously studies have shown that through intracellular signal transduction, TLR ligands in brain tissue activate TLRs (22), mainly TLR-3 and TLR-4 (23), leading to increased secretion of inflammatory factors IFN-γ and IL-17, which directly contribute to the onset of MS. Furthermore, an upregulation of Tyro3, Axl, and Mer receptors, by binding with the extracellular ligand Gas6 (15, 24, 25), promotes the activation of inflammatory inhibitors SOCS1 and SOCS3. It is considered as a negative feedback mechanism and prevents damage that occurs owing to excessive inflammation to regulate the innate immune response (26, 27). Demyelination of periventricular white matter is a typical pathological feature of MS (28). Therefore, we selected the paraventricular tissue as the research object to observe the expression of Gas6, Tyro3, Axl, and Mer in the paraventricular tissue of the Normal, EAE, and Rapamycin groups. Our data revealed that rapamycin could act on the Gas6/TAM pathway, to up-regulate the expression of TAM receptors. Following Gas6/TAM upregulation, there was a significant reduction of TLR3 and TLR4. However, the expression of the anti-inflammatory factors SOCS1 and SOCS3 significantly increased. This upregulation may inhibit the JAK-STAT signaling pathway, which is mediated by IFN-α and promotes inflammation and the differentiation of CD4+T cells to inhibit the secretion of inflammatory factors IFN-γ and IL-17 (29, 30). Rapamycin can also regulate Treg/Th17 cells through the Akt-mTOR and MAPK/ERK signaling pathways (31). Furthermore, it has also been shown to enrich CD4^+^, CD25^+^, CD27^+^, and Foxp3^+^Treg cells (32) in MS patients. IFN-γ is secreted by Th1 cells (33) and IL-17 is secreted by Th17 cells (34). It has been demonstrated that Th1 cells destroy the myelin sheath by secreting cytokines such as IFN-γ, TNF-α, and IL-12 (35, 36). Th17 cells could secrete inflammatory factors (such as IL-17, IL-23, and IL-21) to promote the inflammation (37). Therefore, it seems reasonable to suggest that rapamycin could also inhibit the activation of Th1 cells and the hyper-functionality of Th17 cells. This mechanism could alleviate the damage to the myelin sheath and exert a better immunosuppressive effect to regulate the innate immunity in the pathogenesis of MS. Furthermore, the FoxP3 antibody was used to perform immunostaining to identify the Treg cell population in the brain, more Treg cells were found in EAE group treated with rapamycin (Supplementary Figure 1). It could significantly promote the recovery of neurological symptoms in EAE mice.

The damage to cells in central nervous system (CNS) is measured by intactness and viability of microglia. The functions of microglia are regulated by TAM receptor kinase. The microglial expression of Axl is prominently up-regulated during Parkinson disease (38). Tyro3 was the most widely expressed of the three receptors in CNS, with Axl and Mer detected in only a limited number of sites in the adult (39). Notably, in our research, during the expression of Tyro3, Mer, and Axl, the level of Tyro3 was significantly higher than Mer and Axl. Therefore, this finding suggested that Tyro3 may either have an important role in the negative regulation of EAE or be related to the high expression of Tyro3 mRNA in the CNS. The mechanism underlying the high expression of Tyro3 and the role of TAM and Gas6 expression in different cells types require further research.

In conclusion, our findings suggested that rapamycin can act on the TAM-TLRs-SOCS pathway by inhibiting the secretion of TLR-3, TLR-4, and proinflammatory factors. It alleviates inflammatory demyelination and likely has a neuroprotective role in EAE mice. These findings are significant to understand the role of the TAM-TLRs-SOCS pathway. Furthermore, this study contributes to efforts to elucidate the molecular targets of rapamycin, which is important for the development of immunomodulatory therapies to treat MS.

## Data Availability Statement

The original contributions presented in the study are included in the article/Supplementary Materials, further inquiries can be directed to the corresponding author.

## Ethics Statement

The animal study was reviewed and approved by Lanzhou University Second Hospital.

## Author Contributions

X-lL and M-xW designed experiments. BZ and WL carried out experiments. M-jS, Y-lZ, and HL analyzed experimental results. X-lL wrote the manuscript. M-xW approved the manuscript. All authors read and approved the final manuscript.

## Conflict of Interest

The authors declare that the research was conducted in the absence of any commercial or financial relationships that could be construed as a potential conflict of interest.

## Supplementary Material

Supplementary Figure 1 The numbers of Treg positive cells in the periventricular was analyzed by immunohistochemical analysis. Immunohistochemical analysis of Treg positive cells with anti-FoxP3 antibody at 21 day post immunization (left: Normal group, mid: EAE group, right: Rapamycin group, 200x, 400x magnification). ^**^*P* < 0.01, ^***^*P* < 0.001.
