# Supplementary figures and images for: Rapamycin Alleviates the Symptoms of Multiple Sclerosis in Experimental Autoimmune Encephalomyelitis (EAE) Through Mediating the TAM-TLRs-SOCS Pathway
Source: Front Neurol. 2020 Nov 27;11:590884. doi: 10.3389/fneur.2020.590884 (PMC7728797; doi:10.3389/fneur.2020.590884)

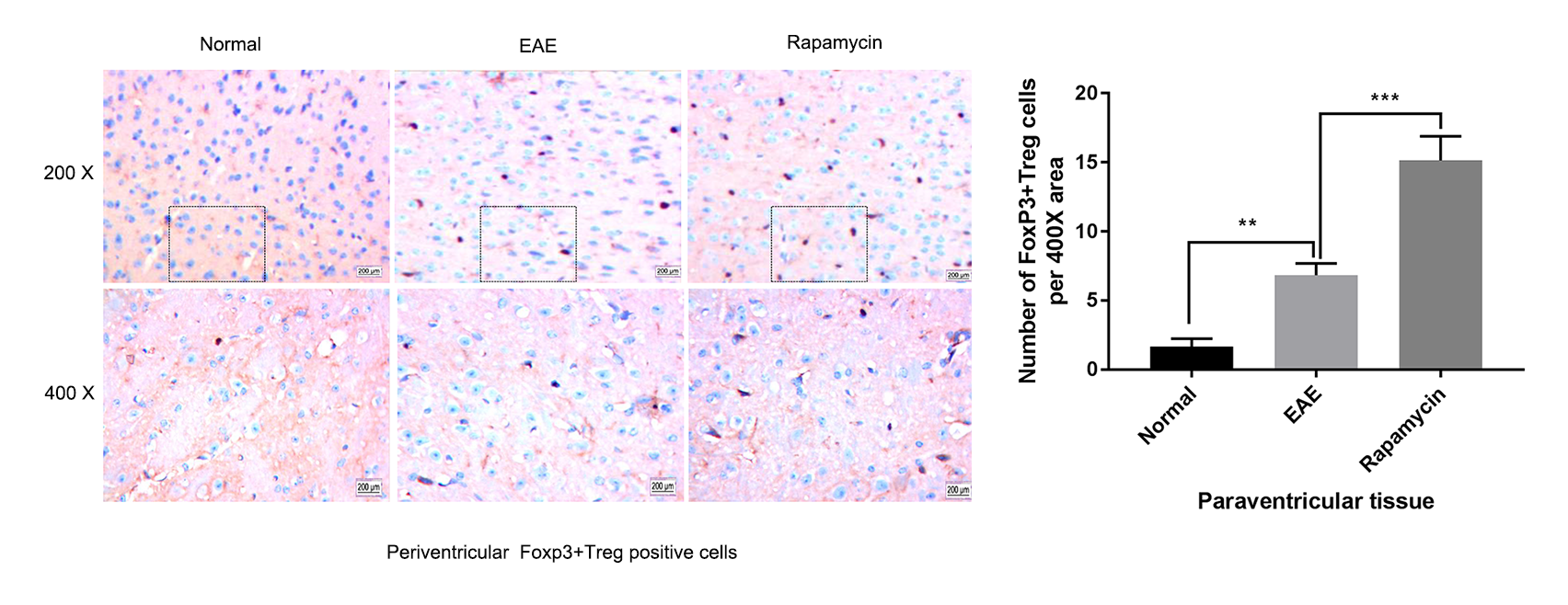

Supplement: Supplementary Figure 1 — The numbers of Treg positive cells in the periventricular was analyzed by immunohistochemical analysis. Immunohistochemical analysis of Treg positive cells with anti-FoxP3 antibody at 21 day post immunization (left: Normal group, mid: EAE group, right: Rapamycin group, 200x, 400x magnification). **P < 0.01, ***P < 0.001. [file Image_1.TIF]
